# Ethical dilemmas in the development of college psychological counseling under the background of cultural integration

**DOI:** 10.3389/fpsyg.2025.1636563

**Published:** 2025-09-10

**Authors:** Wei Xing, Yanan Zheng

**Affiliations:** ^1^School of Humanities and Social Sciences, Gannan Medical University, Ganzhou City, China; ^2^Department of Psychiatry, Gannan Medical University, Ganzhou City, China

**Keywords:** multiculturalism, college psychological counseling, ethical dilemma, the principle of value neutrality, the principle of confidentiality, counseling relationship

## Abstract

A discernible change in the ideological stances of college students has coincided with the growing occurrence of cultural integration in China. At the same time, mental health concerns in higher education have become more prevalent and demand scholarly attention. Inadequate multicultural competency among counselors, unclear professional identity delineation, and inter-regional differences in development are some of the issues facing the current psychological counseling system in higher education institutions that demand immediate attention. Additionally, integrating cultures may require resolving moral conundrums involving principles of value neutrality and confidentiality, among others, which jeopardize the development and upkeep of consultation relationships. To improve the current situation, efforts should be made to enrich the professional guidance and multicultural experience of college counselors, so as to make college psychological counseling adapt to the development and changes of the current situation.

## 1 Introduction

The phenomenon of cultural integration is currently very common throughout China. The most notable examples of cultural blending in higher education are the significant rise in foreign students studying in China and the rising number of students from culturally marginalized backgrounds enrolling on domestic campuses. Empirical research on international students reveals their heightened vulnerability to adverse psychological influences due to the compounded challenges of confronting novel environments, cultural paradigms, and lifestyles (Zhang and Dixon, 2003). However, there is a significantly lower willingness to seek and use psychological counseling services when comparing international student populations to their domestic counterparts (Nilsson et al., 2004). It is imperative that the ethical dilemmas facing psychological counseling services in higher education within culturally blended contexts be urgently examined by scholars in light of the demographic shift toward increasingly diverse student populations. To foster trust relationships with students, establish effective counseling relationships, and safeguard counselee rights appropriately, it imperatively requires addressing pertinent ethical considerations through scholarly discourse and practical resolution.

The “Code of Ethics for Clinical and Counseling Psychology Work of the Chinese Psychological Society” (2018) emphasizes the significance of psychological counselors respecting and acknowledging the cultural diversity of their clients from the dual perspectives of ethical mandates and professional standards. Counselors who are not culturally sensitive may find that their effectiveness is diminished when working with clients from diverse linguistic and cultural backgrounds, primarily due to language problems and a lack of cultural contextualization (Kim et al., 2019). A counselor's ability to acquire and develop accurate cultural perceptual schemas and actively apply them in multicultural counseling processes is known as cultural sensitivity (Ridley et al., 1994). This is the fundamental prerequisite for counselors to develop multicultural competency. Counselors who possess the attitudes, beliefs, knowledge, and abilities necessary to work well with clients from a variety of cultural backgrounds are said to be multiculturally competent (Sue et al., 1992). Multicultural competency among counselors has been shown to increase the efficacy of interventions in clinical settings (Tao et al., 2015). Psychological counselors in China's higher education institutions need to improve their multicultural awareness, according to recent empirical findings (Chen et al., 2023).

From the perspective of interregional developmental disparities, an interview-based study with counseling professionals at two Beijing universities revealed that practitioners proposed a direct concept of “flexibility” in counseling styles. This refers to the ability to adjust counseling approaches according to specific situations, which they deemed crucial for professional development (Che, 2021). Obviously, this is indispensable in multicultural counseling practice. However, surveys conducted with counseling professionals in other regions yielded suboptimal findings, with scant mention of multicultural competence. Surveys of counseling professionals in other areas, however, produced less than ideal results and made very little reference to multicultural competency. For example, there are consistent issues with the counseling staff at Hunan province universities, such as differences in their comprehension and application of confidentiality principles, a lack of hours for supervision, and poor counseling skills (Tang, 2018). North China and East China regions excel in training infrastructure and institutional development due to superior professional standards and strong regulatory frameworks, while Northwest China shows comparative underdevelopment due to lax accreditation criteria for professional institutions and inadequate governance protocols, according to Qian et al. (2008) investigation into psychotherapeutic and counseling administration across six major regions in China.

Furthermore, most Chinese college counselors do not offer full-time student counseling services in accordance with the definition of a professional role. They typically teach a range of subjects, such as mental health education, psychological education, and ideological-political education. Notably, counseling responsibilities are typically performed by part-time staff members, such as student affairs personnel in auxiliary roles. This situation not only results in practitioners lacking professional competence, but it also leaves them overworked and unable to fully commit to their counseling responsibilities. On the other hand, the vague professional designation of psychological counselors in colleges—differently referred to as administrative staff in some and professional technical staff in others—creates role ambiguity that hinders operational implementation and career progression, which in turn reduces the professional appeal of qualified practitioners.

Combining the previously mentioned analysis, there are still noticeable differences between the realities facing psychological counselors in Chinese higher education institutions today and the growing cultural blending phenomena and competency demands on the one hand. In order to provide practitioners working with clients from a variety of cultural backgrounds with operational guidelines and strategic frameworks, this paper outlines its goals to investigate potential ethical conundrums in the development of psychological counseling within higher education settings in culturally blended contexts.

## 2 Ethical challenges faced by college psychological counseling services in the context of cultural integration

### 2.1 Counseling professionals demonstrate inadequate multicultural competence

Chen and Starosta (1996) have proposed that intercultural awareness and intercultural sensitivity correspond respectively to the cognitive and affective dimensions, both playing critical roles in individuals' comprehension of cultural differences. Additionally, they further posit that intercultural awareness constitutes the minimal prerequisite for intercultural competence (Chen and Starosta, 1998). Counselors' multicultural competency encompasses tripartite dimensions of attitudes, beliefs, and knowledge with accompanying skills (Sue et al., 1992), demonstrating fundamental interconnections with practitioners' cognitive and affective domains. It follows that counselors can only fully actualize their multicultural competency when equipped with intercultural awareness and sensitivity, thereby facilitating acute discernment of divergent cultural values between clients and themselves. According to empirical research, practitioners from ethnic minorities exhibit noticeably greater multicultural competency than their mainstream counterparts (Holcomb et al., 2008). From the standpoint of multicultural competency among university counseling professionals, Zeng et al. (2016) multi-regional study in China shows that although practitioners show careful adherence to professional standards, they fall short in applying their skills and improving themselves during counseling sessions. Chen et al. (2023) demonstrate that developing multicultural awareness should be given top priority in order to improve competence, and both structured training participation and experiential accumulation favorably influence its growth. There are currently few studies on multicultural competency among counseling staff at Chinese colleges, and assessment instruments concurrently lack empirical validation of their efficacy. However, current training programs for enhancing faculty members' intercultural communication and pedagogical competence fall short of meeting the demands of international collaboration, according to research on intercultural education in Chinese higher education institutions (Yang and Zainudin, 2024). Developmental counseling techniques are still underutilized in college counseling services, which primarily concentrate on using individual counseling to address students' current psychological problems (Xu, 2020). Counselors' compartmentalized analysis of students' psychological issues, characterized by insufficient attention to their cultural contexts, exacerbates existing insufficiencies in multicultural competency through this fragmented perspective. The professional development of college counseling practitioners is also significantly hampered by China's inadequately developed supervision system for counseling professionals, which is typified by a dearth of organized, useful supervision frameworks (Hou and Zhang, 2007). The American Counseling Association (ACA), on the other hand, expressly requires counselors to learn about cultural diversity and use this knowledge in their work (Barden et al., 2017). This is clearly lacking in China's current frameworks, which denies counseling professionals the institutional motivation to strive for multicultural competency.

Second, in cross-cultural counseling, counselors and clients may encounter the apparent issue of language barriers. Counselor Sentell and his colleagues (2007) have proposed that communication difficulties resulting from limited English proficiency discourage international students from seeking psychological counseling services. Although utilizing interpreter assistance during counseling may potentially alleviate this issue, it simultaneously raises international students' concerns regarding confidentiality, which also deters them from seeking psychological counseling services. International students currently studying in China predominantly employ English as their primary medium of communication within institutional settings. This, in turn, necessitates that counselors possess at least a command of conversational English and demonstrate proficiency in using and explaining specialized psychology terminology in English.

### 2.2 The triple ethical dilemmas of the principle of value neutrality: reliance on authority, conflict of educational goals, and implicit bias

According to Jackson et al. (2013), the neutrality principle in psychological counseling requires counselors to remain impartial, objective, and neutral throughout the therapeutic process while strictly refraining from forcing their own values or belief systems on their clients. The foundational bedrock for trust within the counseling alliance is this principle, which activates clients “autonomy and self-efficacy while preventing misguided interference from counselors” personal values.

Individual autonomy and decision-making are often limited by familial and societal factors in Eastern cultural contexts that emphasize collectivism and peaceful coexistence (Triandis, 2004). Due to cultural tendencies toward deference to authority, clients frequently display elevated expectations for counselors “directive guidance, viewing practitioners as authoritative figures (Duan et al., 2014). Consequently, rigid adherence to value neutrality may precipitate client disengagement within Eastern cultural contexts, as such an approach potentially contravenes culturally embedded expectations of authoritative guidance in therapeutic relationships. Counselors must implement culturally responsive approaches during clinical sessions, adapting the contextual application of the value neutrality principle according to clients” diverse cultural backgrounds.

Comparative research shows that the value neutrality principle is applied differently in Chinese and Western sociocultural contexts when it comes to psychological counseling. According to research, clients and counselors in China expect directive guidance during counseling sessions, and practitioners do in fact frequently give it. However, they predominantly tend to deliver encouraging guidance indirectly while simultaneously concealing this practice (Duan et al., 2014). The value neutrality principle is purposefully upheld by practitioners in Western psychological counseling settings. However, the value systems of clients often converge toward the orientations of their counselors—a phenomenon that takes place without practitioners' conscious knowledge, thus forming an unconscious transfer of counselor values to clients (Kelly, 1990).

When applying the value neutrality principle, Chinese college counseling services must incorporate strong educational orientations. This approach is rooted in institutional educational missions and is closely related to the common dual-role identity of practitioners as both psychological counselors and educators. Therefore, striking a balance between educational missions and value neutrality is a major professional challenge that practitioners in college counseling contexts must carefully consider.

Beyond the aforementioned considerations, the implementation of the value neutrality principle in psychological counseling is further mediated by additional contextual determinants. Scholarly research indicates that counselors must transcend stereotypical constraints to conduct in-depth case exploration (Good and Hannah, 2015). Even ostensibly positive stereotypes may yield detrimental effects when expressed through extreme formulations or disparate cultural lenses, necessitating counselors‘ heightened metacognitive vigilance to counteract instinctual inclinations toward value imposition. Because they supposedly express approval while essentially sustaining depersonalization through categorical generalizations about group characteristics applied to individuals, these stereotypes are classic examples of racial micro aggressions. By weakening the recipients' sense of unique identity, this inferential pattern frequently causes negative psychological reactions (Wang and Guan, 2021). In actuality, cultural biases persist even among the “three forces” of conventional psychological theories. For example, some women and ethnic minorities have expressed dissatisfaction with the psychoanalytic school's treatment of gender concepts (Ivey et al., 1997). Therefore, it remains extremely challenging to entirely eliminate stereotypes, stigmatization phenomena, and their cascading effects within counseling contexts.

To enhance the implementation of value neutrality when engaging with clients from diverse cultural backgrounds, multicultural counseling and therapy theory—recognized as psychology's “Fourth Force”—proposes modifications to traditional prohibitions against counselors providing specific advice or excessive self-disclosure. Given that clients from certain subcultures subjected to discrimination may only explore their issues following counselors‘ substantive self-disclosure, while others culturally expect concrete guidance from practitioners, such prohibitions necessitate strategic relaxation to facilitate therapeutic progress under these specific circumstances (Liu and Ye, 2002). How to ensure adherence to the principle of value neutrality while maintaining respect for clients' cultural backgrounds in situations where counselors are required to disclose personal information, make directive recommendations, or interact with clients who exhibit strong stereotypical labeling is a critical issue that needs to be resolved immediately.

### 2.3 The dual ethical challenges of confidentiality principles: cultural cognitive conflicts and the predicament of mandatory reporting

“Code of Ethics for Clinical and Counseling Psychology Work of the Chinese Psychological Society” (2018) issued by the Chinese Psychological Society mandates that counselors shall maintain full awareness of their personal values and potential impacts on service users, while consistently upholding respect for service users' value systems. Within higher education settings, cultural divergences manifest with particular salience given the diverse cultural backgrounds of student populations contrasted against counselors predominantly originating from mainstream cultural or specialized professional milieus. As manifested in differential orientations toward individualism versus collectivism across Eastern and Western contexts, such variations engender distinct conceptualizations of privacy—disparities that may consequently generate ambiguities for practitioners when determining confidentiality parameters for disclosed information. A documented instance exemplifies how divergent cultural values can precipitate ethical violations (Pedersen, 2007), occurring within a counseling course classroom: an educator renowned in counseling education was formally accused by a student, whose complaint was subsequently referred to the ethics committee of a professional counseling association. The precipitating incident involved the student's cultural discomfort during mandatory counseling coursework with being compelled to disclose personal feelings and activities—perceived as transcending culturally defined boundaries without prior disclosure of this requirement. She initiated dialogue with the professor but perceived her concerns as receiving insufficient attention. The professor reported having consistently maintained identical instructional requirements for other students over the years without encountering previous complaints. The professor maintained that ethical standards mandated uniform treatment of all students, asserting that adjusting pedagogical approaches for cultural backgrounds would constitute inequitable accommodation that could undermine equity for other students. Although the professor expressed regret regarding the student's discomfort, he affirmed his professional obligation to maintain consistent instructional standards. Concurrently, the professor acknowledged that students' cultural differences merit respect as significant considerations. After deliberation during the ethics committee meeting, it was determined that this ethical violation had not been intentional. Furthermore, a measured degree of self-disclosure remains essential within counselor education curricula. However, the professor was instructed to incorporate a formal disclaimer in future course syllabi, signaling that intensive self-disclosure activities may be required. Within the one-on-one counseling process, however, counselors can provide fully individualized therapeutic services tailored specifically to the client. This necessitates counselors' requisite cultural competency to maintain respect for individual cultural differences.

However, counselors in college settings concurrently face ethical dilemmas that necessitate careful navigation. As professional practitioners, counselors are bound by a core ethical mandate requiring rigorous adherence to the principle of confidentiality. However, as institutional educators simultaneously, their professional capacity inevitably necessitates alignment with the college's administrative objectives and institutional stability imperatives. This dual-role configuration generates significant ethical tensions during counseling processes: Counselors may be required to report students “psychological status to school administrators or student cadres; compelled by administrative mandates to disclose disciplinary violations such as academic dishonesty; and under institutional directives, potentially access students” clinical records or disclose non-essential consultation content. Such institutional requisites frequently engender direct conflicts with the confidentiality principle enshrined in counseling professional ethics, constituting infringement upon students “privacy rights. However, beyond college settings, empirical observations of multiple counselors reveal that they do not prioritize the confidentiality principle as a primary obligation within counseling ethical codes. This pattern potentially stems from counselors” disproportionate reactivity to litigation threats coupled with misjudgments in prioritizing client privacy relative to other considerations. Consequently, this practice severely compromises the establishment of therapeutic alliances and impedes counseling progress. Scholars consequently advocate that counselors must withstand institutional pressures, disclosing only the minimally essential information when confidentiality exceptions become unavoidable (Donner et al., 2008).

### 2.4 The dual challenges in establishing a counseling relationship: cultural barriers and communication deviations

In counseling settings, different cultural value systems can lead to misunderstandings and disputes between clients and counselors. Cultural differences in speech patterns and nonverbal clues can greatly reduce communication effectiveness, and counselors who lack cultural sensitivity run the risk of failing to fully understand the needs and emotional states of their clients. The complexity of building a successful therapeutic rapport in multicultural counseling settings is increased by these compounding factors taken together. Additionally, the development of trust between clients and counselors is hindered by cultural barriers. The intricacy of developing therapeutic rapport is increased when cultural misunderstandings result in professional errors that fail to appropriately understand or address clients “psychological concerns. These errors also increase skepticism toward practitioners” clinical competence. Clients prefer counselors who share their personality traits, according to empirical research (Anestis et al., 2020). Therefore, in order to attain the best possible therapeutic alignment, practitioners should make specific modifications to the counseling process when working with clients who have different cultural backgrounds or who have different personality traits.

In summary, ethical principles encompassing professional competency, value neutrality, privacy and confidentiality standards, and therapeutic alliances confront novel challenges within cultural integration contexts, as schematized in Table 1.

**Table 1:** Ethical principles of psychological counseling and corresponding multicultural challenges.

**Ethical principles of psychological counseling**	**Multicultural challenge**
Professional competence	Multicultural competence
	Language communication disorder
The principle of value neutrality	The matching of cultural background and values
	The constraints of stereotypes or stigmatization
	The conflict in the orientation of educational goals
Privacy and confidentiality issues	The matching of cultural background and privacy view
	The influence of personal prejudice
	The conflict between management goals and confidentiality
Consultation relationships	The matching of cultural background and language expression habits
	The establishment of trust in times of cultural estrangement

## 3 Discussion

College counseling in the framework of cultural integration is the main topic of this study. Interregional developmental disparities, a lack of qualified practitioners, and a lack of professional competence among current counselors are just a few of the major issues facing college counseling systems today. In light of these problems, the phenomenon of cultural convergence may present serious obstacles to ethical standards such as the management of consulting relationships, value neutrality principles, and confidentiality principles. These conundrums arise from the conflict and blending of cultural differences and, more importantly, entail striking a balance between ethical integrity and cultural diversity. Building upon these findings, the following practical recommendations are proposed.

### 3.1 Building a supervision system from a multicultural perspective

Clinical supervision systems must be put in place before psychological counseling and psychotherapy can become professionally recognized (Bernard and Goodyear, 2005), and supervision is a crucial part of helping new practitioners become more competent. According to empirical research, college counselors who have participated in more training and have worked in clinical settings for a longer period of time are much more multi culturally competent (Chen et al., 2023). This implies that multicultural skills will gradually improve in tandem with the amount of counseling experience gained. In addition to facilitating a thorough examination of counselors' case experiences, supervision serves as a professionalized guidance mechanism that transfers experiential knowledge from seasoned practitioners to trainees.

Second, from the standpoint of knowledge and skill iteration and continuous update, supervision training must impart new counseling knowledge and skills that are consistent with the history of the present era. Counselors should learn how to work flexibly with clients from diverse cultural backgrounds and develop their cultural sensitivity, just as they would in the context of integrating multiple cultures. It assists counselors in putting their multicultural knowledge and abilities to use in their real work through case discussions, role-playing, simulated counseling, etc. A model of supervision has been developed by academic research, offering a thorough examination of its purposes, theoretical underpinnings, and stages of development (Körük and Kara, 2019). It is clear that the supervision and training needed to improve counselors' multicultural competency should be a modern, adaptable professional procedure.

Concurrently, a supervisory mechanism for ethical compliance auditing must be established within psychological counseling processes, mandating rigorous disciplinary measures against practitioners violating ethical standards. When counselors are unable to ensure adherence to ethical protocols, clients shall be systematically referred to qualified professionals capable of providing appropriate professional services. The establishment of such supervisory mechanisms not only safeguards client rights but also constitutes a critical safeguard for ensuring service quality in psychological counseling within multicultural contexts.

This domain, which focuses on the clinical supervision systems in place in China, shows a notable lack of maturity. First and foremost, China has a serious shortage of skilled workers with the credentials to provide supervision training in the country. In addition to meeting master's or doctoral degree requirements, credentialed supervision staff must also have a significant amount of clinical experience. Very few practitioners currently meet both requirements. This disparity results from China's overemphasis on theoretical education in comparison to real-world application in counselor development. Even though there are many short- and long-term psychological counseling training programs across the country, trainees usually don't have enough opportunities for real-world application. Additionally, many graduate students in China are subjected to coursework that is solely focused on writing theses, routinely ignoring real-world internship experiences (Hou and Zhang, 2007). As such, it demands that practice-oriented pedagogical paradigms be strengthened. In particular, it is advised that graduate education systems be reformed to make graduation requirements for psychology-related programs include standardized, long-term practicum components. At the same time, systematic longitudinal supervisor training programs focused on improving practical competency should be developed. The current gap in access to qualified supervision, however, might be reduced by creating specialized supervisory resource coordination platforms that would encourage professional interaction among certified supervisors and encourage them to offer professional supervisory services to new counselors.

### 3.2 Enrich the multicultural experiences of college counselors to enhance their multicultural competence

Empirical investigations into multicultural competency among Chinese university counselors reveal that despite substantive knowledge acquisition regarding multicultural counseling, practitioners frequently demonstrate deficient practical consciousness in application contexts, indicating considerable difficulty in translating knowledge into behavioral implementation (Chen et al., 2023). According to studies on multicultural experiences, leaders who have been exposed to different cultures are better able to take on different viewpoints when facing difficulties, as well as being more resilient and independent under pressure. These traits have a positive impact on their capacity for creativity, problem-solving, and self-control. Additionally, by influencing personal characteristics and encouraging cognitive flexibility, multicultural encounters improve people's metacognitive capacities (Li et al., 2022). In order to empower clients to acquire problem-solving skills, psychological counseling is a process that requires self-regulation on the part of both clients and practitioners. Additionally, metacognitive processes allow counselors to assess the objectivity and validity of their views toward clients, which is a crucial element that is especially important in cross-cultural counseling settings.

Regarding concrete methodologies for enhancing counselors' multicultural experiential development, while no scholarly investigations have specifically examined the current landscape and challenges of multicultural experiences among Chinese psychological counselors, international research has identified immersive multicultural experiences as an empirically validated approach to significantly enhance multicultural competency (Barden and Cashwell, 2013). The main approach entails students actively participating in a foreign cultural setting and working with a designated cultural liaison to talk about their cultural values and viewpoints. All cognitive-affective experiences, both positive and negative, must be recorded by participants during the process, which ends with open-ended classroom debriefing sessions. This emphasizes that the most direct and efficient ways to improve cultural sensitivity and multicultural competency are through first-hand experiences and methodical documentation.

Implementing immersive multicultural experiences within China's cultural confluence context necessitates counselors' entry into interactive settings with culturally minoritized groups, such as providing access platforms to ethnic enclaves to facilitate immersion in diverse cultural lifestyles and value systems. This experiential engagement is augmented through structured dialogues with cultural liaisons (e.g., ethnic representatives), enabling cognitive processing of intercultural dissonance while systematically deriving culturally adaptive counseling strategies via critical reflection. Following each immersive encounter, counselors are mandated to contemporaneously document salient incidents within bidirectional cultural interactions, with particular attention to cataloging the nature and intensity of resultant affective experiences. Subsequently, counselors engage in structured open dialogues with fellow practitioners, representatives of the relevant minoritized cultural groups, and clinical supervisors. These discussions encompass reconstructing cultural encounter scenarios, analyzing value-based origins underlying behavioral discrepancies, collaboratively formulating culturally adaptive intervention frameworks, and conducting simulated role-playing exercises to empirically evaluate intervention efficacy from client perspectives.

### 3.3 The institutional reconstruction of psychological counseling in colleges and the promotion of ethical compliance

First, college students' psychological problems resulting from negative emotions have gotten worse recently (Gao et al., 2020), which means that higher education institutions need to pay more attention to them and provide targeted interventions. Counselors in higher education institutions are obviously important because they are the main people protecting college students' mental health. In order to reduce the prevalence of dual relationships, accredited higher education institutions should give top priority to hiring qualified, full-time counselors. They create a strong basis for establishing fruitful consulting relationships and applying value neutrality concepts in clinical practice by doing this. Second, in order to guarantee greater independence, counseling centers in higher education institutions should remain clearly isolated from other institutional entities. This structural difference strengthens protections for client privacy and makes it easier to adhere to confidentiality principles more strictly.

Furthermore, by integrating pertinent cultural customs, institutions can create individualized counseling plans for students from a variety of cultural backgrounds once they have established strong faculty resources. This customized strategy guarantees that students receive better, culturally sensitive interventions while also increasing the effectiveness of the counseling process. However, to avoid unfavorable results from privacy violations, the plan development process necessitates meticulous attention to gaining students' informed consent and rigorous adherence to confidentiality principles.

Students undergoing academic migration to cross-cultural environments may encounter systemic barriers such as geographical separation and time zone disparities post-return, substantially compromising the continuity of sustained psychological counseling interventions. According to Wu et al. (2020), objective barriers like scheduling conflicts and long wait times account for 15% of client discontinuations, indicating that counseling attrition is still a major problem in college settings. In order to meet students‘ demands for flexible scheduling and multi-location accessibility, college counseling centers ought to think about introducing technology-based online counseling platforms. This will improve the effectiveness and convenience of psychological support services. Research has documented that mental health service platforms in China demonstrate a significantly higher proportion of negative lexicon and lower frequency of positive expressions in professional responses compared to their international counterparts, which exhibit markedly inverse lexical patterns. This suggests discernible divergences in the utilization of supportive strategies and techniques across distinct cultural contexts (Zhang et al., 2024). Consequently, counselors must continuously maintain cultural awareness of clients' backgrounds and employ culturally adaptive strategies even when delivering services through online platforms. Besides, the effectiveness of counseling or the application of confidentiality principles may be jeopardized by online counseling. To guarantee their correct implementation and protect service integrity, strong platforms and institutional regulations are therefore essential.

Group counseling has been found to be an effective intervention modality for improving psychological well-being and facilitating environmental adaptation in empirical research on international students in China. Participants in group counseling interventions led by Chinese scholars with international students in China have reported feeling more a part of the community, feeling more supported by others, and then adopting more flexible coping mechanisms (Liu et al., 2022). To increase international students' sense of social support and improve their psychological well-being, Chinese higher education institutions may include group counseling in their mental health curricula for overseas students, plan frequent structured interventions, and think about including domestic peers in group settings.

### 3.4 Promote interdisciplinary collaboration to enhance multicultural service capabilities

In order to understand psychological distress, researchers have suggested that mental health practitioners should use an anthropological perspective. They contend that it is important to place mental health concerns in larger sociocultural contexts rather than limiting interpretations to specific psychopathological frameworks (Kuah-Pearce et al., 2014). As a result, one psychological viewpoint is insufficient to fully comprehend a person's psychological problems, especially in cross-cultural settings. The traditional “three forces” of psychological theories also reflect this limitation, as most counseling and psychotherapy theories focus on individual identity frameworks and pay little attention to collective dimensions (Li, 2009). The creation of interdisciplinary research teams with specialists in psychology, sociology, anthropology, education, and related disciplines is necessary to meet this challenge. This calls for creating long-lasting cooperative systems to jointly explore the theoretical and practical aspects of counseling in multicultural settings, carrying out cooperative research projects, and eventually producing integrated instructional materials.

To advance interdisciplinary intellectual exchange and research collaboration in response to demands for enhanced multicultural service competency, establishing multidisciplinary collaborative assessment teams may effectively address cases involving complex sociocultural factors. The core composition of such teams may encompass psychologists, social workers, anthropologists, and educational specialists, with flexible inclusion of relevant stakeholders such as parents when context-specific needs arise. The team must initially convene preliminary meetings to delineate assessment dimensions, thereby ensuring comprehensive case evaluation. Subsequently, core members conduct specialized assessments according to their professional divisions of labor. Following the completion of disciplinary analyses, the full team reconvenes for integrative synthesis sessions wherein all members pool findings, engage in in-depth deliberation, and explicate the interwoven influences among individual symptoms and social stressors, cultural contexts, interpersonal dynamics, and educational systems. The team collaboratively formulates comprehensive intervention plans during final deliberations, ensuring inclusion across psychological counseling, family work, school-based education, and community resource systems. Most critically, this joint assessment process must be underpinned by explicit confidentiality protocols and regular interdisciplinary case supervision to safeguard both the professional integrity of core members and confidentiality assurance for individual cases.

### 3.5 Promoting continuing education on consultation ethics for college counselors

For college counseling professionals, continuing education becomes especially important in the context of cultural integration. It is a vital part of bolstering ethical education in addition to providing a means of improving professional competencies and specialized knowledge. Professionals who have finished their foundational education, are actively practicing their trade, and have adult responsibilities are the target audience for continuing education. However, there are significant gaps in the structure and content of the college counseling professionals' current continuing education programs. Research by Qian et al. (2009) reveals that practitioners in China's psychological counseling sector, including school-based counselors, collectively demonstrate persistent deficiencies in professional ethics training, with particularly pronounced gaps in competencies pertaining to counseling alliances and confidentiality principles. They also don't know how to ask for help when faced with ethical dilemmas in their line of work. Therefore, it is essential to explicitly stipulate the completion of specific instructional hours in ethics training within industry regulatory frameworks and to legally mandate continuing education in ethical guidelines in order to ensure the ongoing advancement of professional ethical standards. Additionally, when registering counseling-related certifications and evaluating professional titles for practitioners, counseling ethics must be given the consideration it deserves.

### 3.6 Specific implementation steps to improve the development dilemma of college consulting

Researchers have previously proposed the Cognitive Skills Supervision Model for counseling (Körük and Kara, 2019), which incorporates instructional components delivered by supervisors to counselors within its framework. The model is fundamentally structured across four sequential phases: The initial phase focuses on cultivating cognitive awareness, wherein counselors must undertake literature reviews to enhance theoretical knowledge while critically examining their own cognitive processes. The second phase involves supervisors introducing counselors to three fundamental competencies: information gathering, hypothesis formulation, and intervention planning—establishing foundational skills for subsequent clinical application. Transitionally, the third phase centers on counselors' cognitive self-assessment, marking the model's operational shift toward practice-oriented development. Counselors are required to conduct cognitive processing under supervisory guidance, reapply these cognitive competencies within clinical sessions, and subsequently scrutinize their professional performance. The culminating phase involves cognitive skill implementation, wherein counselors are required to operationalize competencies acquired throughout the preceding three stages. These applications may be conducted across diverse practice modalities such as clinical sessions, role-playing exercises, or case analysis scenarios. Researchers have posited that this cognitive skills supervision model inherently necessitates particular attention to cultural factors, as diverse cultural backgrounds fundamentally shape cognitive formation processes. During the initial two phases, counselors may enhance their cross-cultural counseling proficiency by engaging with scholarly literature, acquire explicit command of fundamental skills through instruction from seasoned supervisors, and foster cognitive restructuring pertinent to multicultural contexts. Through subsequent experiential application and deliberate reflection, theoretical constructs of intercultural counseling become internalized as practical skills mastery. Thus, this clinical supervision model demonstrates substantive utility within contexts of multicultural confluence.

However, this clinical supervision model presents potential limitations: While emphasizing cognitive analysis, it inadequately addresses non-cognitive dimensions such as racially triggered affective experiences arising from multicultural conflicts—challenges insufficiently mitigated through skill-based training protocols. Furthermore, should counselors possess inadequate cultural comprehension regarding specific backgrounds, this supervision model risks reinforcing preexisting stereotypes about particular groups. More critically, it may engender novel yet reductive stereotypes toward certain populations—developments fundamentally incompatible with maintaining counselors' adherence to value neutrality principles. Ultimately, this supervision model imposes substantial demands on supervisors' multicultural competency, requiring them not only to facilitate supervisees' identification of underlying cognitive issues but also to demonstrate pedagogical proficiency in instructing cross-cultural counseling techniques—a dual responsibility that constitutes a significant professional challenge for supervisors themselves.

Assessment of counselors' multicultural competency currently suffers from scarce domestic research, with only a limited number of scholars having undertaken translation and adaptation of existing international scales. Ponterotto et al. (2002) developed the Multicultural Counseling Knowledge and Awareness Scale (MCKAS). This instrument comprises 32 items employing a self-report methodology to respectively assess two distinct dimensions: multicultural counseling knowledge and multicultural awareness. Employing a 7-point Likert scale, higher scores indicate respondents' greater knowledge and awareness in multicultural counseling. The scale demonstrates sound psychometric properties, including established reliability and validity. Subsequently, Chen et al. (2023) conducted indigenization and translation adaptations aligned with Chinese cultural contexts, eliminating three items culturally incongruent with Chinese contexts. The scale maintained satisfactory internal consistency reliability throughout this adaptation process. However, since this scale constitutes a modified adaptation of an existing Western instrument with culturally embedded content, such truncation may result in the omission of critical indigenous dimensions while simultaneously failing to encompass China-specific thematic elements, thereby diminishing the scale's cultural sensitivity within local contexts. Consequently, developing a professionally validated evaluation instrument for counselors' multicultural competency that is culturally congruent with Chinese contexts represents a critically urgent imperative.

To sum up, this study methodically looks at the moral conundrums that arise when college counseling services are developed in the framework of cultural integration. Three main contradictions across critical dimensions are identified in this study based on an analysis of current conditions and issues. First, college counselors' present multicultural competency falls short of what is needed in culturally integrated settings. Second, cultural differences lead to disagreements about value neutrality and confidentiality principles in counseling. Third, the development of consultation relationships is severely hampered by cultural differences. To address these challenges, this paper proposes strategic interventions including the development of a multicultural-perspective supervision system and the innovation of institutional mechanisms for college counseling services. These suggestions offer a theoretical framework for developing a counseling system that is sensitive to cultural differences in multicultural environments. In essence, resolving such ethical dilemmas requires consistent work to strike a balance between cultural sensitivity and the professionalization of psychological counseling, as well as to foster interdisciplinary collaborative innovation in psychology, ethics, anthropology, and related fields.
